# Stage A Heart Failure Is Not Adequately Recognized in US Adults: Analysis of the National Health and Nutrition Examination Surveys, 2007-2010

**DOI:** 10.1371/journal.pone.0132228

**Published:** 2015-07-14

**Authors:** Lara C. Kovell, Stephen P. Juraschek, Stuart D. Russell

**Affiliations:** 1 Division of Cardiology, Department of Medicine, Johns Hopkins University School of Medicine, Baltimore, Maryland, United States of America; 2 Department of Epidemiology, Johns Hopkins Bloomberg School of Public Health, Baltimore, Maryland, United States of America; Mayo Clinic, UNITED STATES

## Abstract

**Background:**

Stage A heart failure (HF) is defined as people without HF symptoms or structural heart disease, but with predisposing conditions for HF. This classification is used to identify high risk patients to prevent progression to symptomatic HF. While guidelines exist for managing HF risk factors, achievement of treatment goals in the United States (US) population is unknown.

**Methods:**

We examined all adults with Stage A HF (≥20 years, N =4,470) in the National Health and Nutrition Examination Surveys (NHANES) 2007-2010, a nationally representative sample. Stage A HF was defined by coronary heart disease (CHD), hypertension, diabetes mellitus, or chronic kidney disease. We evaluated whether nationally accepted guidelines for risk factor control were achieved in Stage A patients, including sodium intake, body mass index, hemoglobin A1c (HbA1c), cholesterol, and blood pressure (BP). Pharmacologic interventions and socioeconomic factors associated with guideline compliance were also assessed.

**Results:**

Over 75 million people, or 1 in 3 US adults, have Stage A HF. The mean age of the Stage A population was 56.9 years and 51.5% were women. Seventy-two percent consume ≥2g sodium/day and 49.2% are obese. Of those with CHD, 58.6% were on a statin and 51.8% were on a beta-blocker. In people with diabetes, 43.6% had HbA1c ≥7%, with Mexican Americans more likely to have HbA1c ≥7% . Of those with hypertension, 30.8% had a systolic BP ≥140 or diastolic BP ≥90 mm Hg. Having health insurance was associated with controlled blood pressure, both in those with hypertension and diabetes. In CHD patients, income ≥$20,000/year and health insurance were inversely associated with LDL ≥100mg/dL with prevalence ratio (PR) of 0.58 (*P*=0.03) and 0.56 (*P*=0.03), respectively.

**Conclusions:**

One-third of the US adult population has Stage A HF. Prevention efforts should focus on those with poorly controlled comorbid disease.

## Introduction

Over 5.1 million Americans have heart failure (HF), a devastating disease associated with high morbidity, mortality, and cost.[1] Despite advances in therapies, only 50% of patients survive 5 years after diagnosis with HF.[2] The estimated cost of HF was $32 billion in 2013 with projections predicting $70 billion by 2030.[1,3] In an attempt to encourage prevention and identify people at risk for HF, the American College of Cardiology (ACC)/American Heart Association (AHA) defined Stage A HF as people without HF symptoms but with related comorbid diseases conferring high risk for developing HF, such as coronary heart disease (CHD), diabetes mellitus, hypertension, or chronic kidney disease.[4] However, adequate control of risk factors among US adults with Stage A HF is unknown.

While prevention has been the focus of CHD and diabetes for decades, HF prevention lags behind with new estimates suggesting 1 in 33 people will have HF by 2030.[3] HF prevention involves adjusting lifestyle factors such as salt intake and weight, risk factor control (body mass index, blood pressure, cholesterol, blood sugar), and the use of medications proven to curb progression to HF.[5–16] While aggressive risk factor control is recommended in Stage A HF to prevent HF progression, the degree of control and rates of therapy in this population, along with the factors associated with risk reduction, have never been determined in the US population.

The purpose of the present study was to characterize US adults with Stage A HF in a nationally representative population, using the National Health and Nutrition Examination Survey (NHANES) conducted from 2007–2010. Additionally, we examined the achievement of risk factor control, the use of pharmacologic interventions with known efficacy in delaying HF onset, and the demographic and socioeconomic variables associated with medication use and poor control of risk factors.

## Methods

### Study Population

The NHANES are cross-sectional studies conducted by the National Center for Health Statistics (NCHS). The study population was recruited via mobile examination centers, which visit US communities selected using a complex, multistage sampling design to represent the age, sex, and racial/ethnic distribution of the US. In this study, we used the interviews, physical examinations, and laboratory measurements gathered by the continuous NHANES surveys (2007–2010) from participants age 20 years and older. These studies were approved by the NCHS Research Ethics Review Board and informed consent was obtained from all participants.[17]

### Stage A Heart Failure

Stage A HF was defined among participants without a diagnosis of heart failure as any of the following self-reported conditions that were identified by a doctor or other health professional: CHD/myocardial infarction (MI), diabetes mellitus, hypertension, or chronic kidney disease. We used self-report rather than laboratory or anthropometric measures because these represented a population for which a physician or health professional had identified a Stage A HF condition.

### Heart Failure Risk Factors

We examined risk factors known to be associated with the progression of HF that also had well-established treatment guidelines. Dietary sodium intake was determined via 24-hour dietary recall interviews, initially administered in-person then followed by a telephone call 3–10 days later. Sodium intake was dichotomized as <2000 mg/d and ≥2000 mg/d.[18] Body mass index (BMI) was determined by measuring standing height and weight and stratified by obese (BMI ≥30 kg/m^2^) or not obese (BMI <30 kg/m^2^). Low density lipoprotein cholesterol (LDL) was estimated in a subsample of NHANES participants using the Friedewald equation[19] and dichotomized according to ATP III guidelines (<100 mg/dL vs. ≥100 mg/dL) with an alternative guideline cutpoint of 70 mg/dL as well.[20] Hemoglobin A1c was dichotomized as <7% or ≥7%. Control of blood pressure was determined by systolic blood pressure (SBP) <140 mm Hg and diastolic blood pressure (DBP) <90 mm Hg among persons with a prior diagnosis of hypertension.[21] We examined two targets for blood pressure control in participants with a prior diagnosis of diabetes or chronic kidney disease based on the more stringent JNC VII (SBP <130 and DBP <80 mm Hg) or the 2014 Hypertension guidelines (SBP <140 and DBP <90 mm Hg).[21,22] Smoking status was examined in categories of never, former, or current. Alcohol use was examined in categories of never, former, non-excessive current, or excessive current.[23]

### Demographic and Socioeconomic Factors

Age, sex, and race/ethnicity information were collected from all participants. Race/ethnicity was characterized as non-Hispanic white, non-Hispanic black, Mexican American, Hispanic, and Other.[17] Socioeconomic factors examined were family income, health insurance, and education level. Self-reported annual family income data was collected from study participants and dichotomized as ≥$20,000 or <$20,000. Health insurance coverage was based on an affirmative response to the following question: “Are you covered by health insurance or some other kind of health care plan?” Education level was ascertained with the question “What is the highest grade or level of school you have received?” Educational attainment was subsequently dichotomized as ≥ some college or < some college.

### Medication Use

Participants were asked to bring their medication bottles to the examination center. Medications used in the past 30 days were recorded by NHANES staff. In this study, we grouped medications in the following three categories: angiotensin converting enzyme (ACE) inhibitors or angiotensin II receptor antagonists (ARBs), HMG-CoA reductase inhibitors (statins), or beta-adrenergic blocking agents (beta-blockers).

### Statistical Analyses

All analyses were performed using the sample weights, primary sampling units, and strata in concordance with NCHS recommendations to account for the NHANES complex sampling design.[24] Standard errors were determined for all metrics using the Taylor series (linearization) method. Prevalence ratios were estimated via Poisson regression, which provides a more accurate estimate of prevalence than logistic regression.[25] Analyses were performed with Stata 11.1 (StataCorp LP, College Station, TX).

Characteristics of NHANES participants with Stage A HF were determined using weighted means or prevalence estimates along with standard errors. We also evaluated the prevalence and number of US adults with Stage A HF and achievement of risk factor targets. Poisson regression and prevalence ratios were used to examine the association between family income ≥$20,000, health insurance, or education ≥ some college and the achievement of recommended risk factor targets. Poisson models were nested as follows. Model 1 was adjusted for age, sex, and race/ethnicity, while Model 2 was adjusted for Model 1 and family income, health insurance status, and education level. We also explored demographic factors associated with meeting risk factor targets, adjusting for Model 2 covariates and using forest plots.

In order to maintain consistency with the CHD/MI category, the estimates for LDL were made using the standard 2-year weights rather than the recommended subsample weights. A sensitivity analysis evaluating the LDL using the recommended weighting scheme yielded virtually identical results.

## Results

Table 1 shows recommendations for lifestyle modifications and treatment targets for Stage A HF, based on guidelines available during the survey period 2007–2010 as well as current guidelines.[20–22, 26–32] There were 11,730 adults, age 20 and older, surveyed and examined at the mobile examination center in NHANES 2007–2010. Population characteristics are in Table 2. The number of US adults in 2007–2010 with Stage A HF was over 75 million, corresponding to 33% of the US adult population (Table 3).[33] Examination of modifiable risk factors found that sodium intake was >2,000 mg of salt per day in 72% of these participants. Nearly half were obese with an additional 32% overweight. Among adults with CHD or prior MI, 36.3% had LDL ≥100 mg/dL and 78.5% had LDL ≥70 mg/dL. In Stage A HF patients with diabetes, the prevalence of HbA1c >7% was 44%. Furthermore, 49.2% had a SBP ≥130 or DBP ≥80 mm Hg while 26.9% had a SBP ≥140 or DBP ≥90 mm Hg. Of those with hypertension, 30.8% had a SBP ≥140 or DBP ≥90 mm Hg. In those with kidney disease, 40.0% had SBP ≥130 or DBP ≥80 mm Hg while 18.2% had SBP ≥140 or DBP ≥90 mm Hg. With regards to medications, in those with CHD/MI, only 45.9% were taking ACE inhibitors/ARBs, 58.6% were taking statins, and 51.8% were taking beta-blockers. ACE inhibitor/ARB use was also determined in participants with diabetes, of whom 50.8% were taking this class of medications.

**Table 1:** Recommendations for Treatment Targets in Stage A Heart Failure.

	Target/Goal	Guideline
**Lifestyle Factors**		
Daily sodium intake	< 1500 mg/d*	AHA, HHS, USDA
	< 2300 mg/d†	
	No target	IOM
Weight	BMI < 30 kg/m^2^	ACC/AHA
	BMI < 25 kg/m^2^ ‡	ACC/AHA
Tobacco use	Advise about hazards, encourage to quit	ACC/AHA
Alcohol use	Counsel about intake	ACC/AHA
Exercise	150 minutes per week, moderate intensity	CDC, ACC/AHA
**Control of Co-morbid Disease**		
**Diabetes**		
Glycemia	HbA1c < 7.0%	ADA
Blood pressure	SBP<140 mm Hg, DBP<90 mm Hg	ADA 2014, 2014 Hypertension guidelines
	SBP<130 mm Hg, DBP<80 mm Hg	ADA 2013, JNC VII
Lipid management	LDL<100 mg/dl, LDL<70 mg/dl‡	ADA 2014, NQF
	Moderate to high intensity statin, if age < 75 years, based on 10-year ASCVD risk	ACC/AHA 2013
ACE inhibitor/ARB	Start if Albumin:creatinine ratio ≥30 mg/g	ADA
**Hypertension**		
Blood pressure	SBP<150 mm Hg, DBP < 90 if age ≥ 60 years	2014 Hypertension guidelines
	SBP<140 mm Hg, DBP<90 mm Hg if age < 60 years	2014 Hypertension guidelines
	SBP<140 mm Hg, DBP<90 mm Hg	JNC VII
**CHD/MI**		
Lipid management	LDL<100 mg/dl, LDL<70 mg/dl‡	ATP III
	High intensity statin if age < 75 years	ACC/AHA 2013
ACE inhibitor/ARB	Consider for all patients	ACC/AHA
Beta-blocker	Start and continue indefinitely	ACC/AHA
Statin	To achieve goals as above	ACC/AHA
**CKD**		
Blood pressure	SBP<140 mm Hg, DBP<90 mm Hg	2014 Hypertension guidelines
	SBP<130 mm Hg, DBP<80 mm Hg	JNC VII, NKF
ACE inhibitor/ARB	Start if Protein:creatinine ratio ≥200 mg/g	NKF

* ≥51 years old, African American, HTN, DM, CKD

^†^ General population

^‡^ Ideal target

Abbreviations: BMI: body mass index; AHA: American Heart Association; HHS: US Department of Health and Human Resources; USDA: US Department of Agriculture; IOM: institute of medicine; BMI: body mass index; ACC: American College of Cardiology Foundation; CDC: Centers for Disease Control; HbA1c: hemoglobin A1c; ADA: American Diabetes Association; NQF: National Quality Forum; SBP: systolic blood pressure; DBP: diastolic blood pressure; JNC: Joint National Committee; CHD: coronary heart disease; MI: myocardial infarction; LDL: low-density lipoprotein cholesterol; ATP: Adult Treatment Panel; ACE, angiotensin converting enzyme; ARB, angiotensin receptor antagonists; Statin, HMG-CoA reductase inhibitors; Beta-blocker, beta-adrenergic blocking agent; CKD: chronic kidney disease; NKF: National Kidney Foundation

**Table 2:** Population Characteristics of US Adults ≥20 With and Without Stage A HF, 2007–2010.

	No HF	Stage A HF	HF	Overall
	Weighted mean (SE) or %			
Age, years	41.3 (0.3)	56.9 (0.33)	66.0 (0.7)	46.9 (0.3)
Male, %	47.9	48.5	53.4	48.2
Race/Ethnicity, %				
Non-Hispanic white	67.5	70.9	69.0	68.7
Non-Hispanic black	10.0	13.5	17.1	11.3
Mexican American	9.9	5.9	3.3	8.5
Hispanic	5.5	4.0	3.6	5.0
Other	7.0	5.6	7.0	6.6
Annual family income ≥$20,000, %	83.0	80.3	72.3	81.9
Highest level of education ≥high school, %	59.1	52.4	39.2	56.4
Participant possesses health insurance, %	75.6	87.8	94.0	79.9
Body Mass Index (kg/m^2^)	27.4 (0.1)	30.9 (0.1)	31.9 (0.5)	28.6 (0.1)
Hemoglobin A1c, %	5.4 (0.01)	6.0 (0.03)	6.4 (0.1)	5.6 (0.02)
Systolic blood pressure, mm Hg	117.1 (0.3)	129.4 (0.3)	126.1 (1.3)	121.4 (0.3)
Diastolic blood pressure, mm Hg	69.8 (0.4)	71.0 (0.4)	64.2 (0.9)	70.1 (0.4)
High density lipoprotein cholesterol, mg/dL	53.6 (0.4)	50.7 (0.4)	48.2 (1.1)	52.6 (0.3)
Low density lipoprotein cholesterol, mg/dL	118.2 (0.8)	112.2 (1.0)	105.9 (4.5)	116.0 (0.7)
Estimated GFR (mL/min per 1.73m^2^)	100.8 (0.6)	85.0 (0.6)	69.4 (1.9)	95.0 (0.5)
MI/CHD, %	0	11.2	56.7	4.9
Diabetes, %	0	25.0	41.3	9.1
Hypertension, %	0	87.6	75.6	30.3
Kidney Disease, %	0	5.0	16.8	2.0
Sodium intake, mg	3,653 (33)	3,398 (48)	2,887 (98)	3,552 (29)
Smoking status, %				
Never	56.9	49.9	40.1	54.3
Former	19.7	31.9	42.2	24.2
Current	23.4	18.1	17.7	21.5
Alcohol use, %				
Never	10.0	13.4	16.0	11.3
Former	18.8	29.8	44.1	23.0
Non-excessive current	35.7	36.3	29.7	35.8
Excessive current	35.5	20.4	10.2	30.0

Abbreviations: HF, heart failure; GFR, glomerular filtration rate; MI, myocardial infarction; CHD, coronary heart disease

**Table 3:** Control of Risk Factors and Medication Use in US Adults≥20 with Stage A HF.

	Weighted No. (in millions)	Proportion of Weighted No. (%)	US prevalence*
**All persons with Stage A HF**	75.3		33.35 (0.76)
*Sodium intake (mg/d)*			
<2000	13.4	19.8	
≥2000	54.2	80.2	
*Body Mass Index (kg/m^2^)*			
<25	12.9	18.5	
25–29.9	22.4	32.3	
≥30	34.2	49.2	
**Persons with CHD/MI**	7.74		3.66 (0.21)
*Cholesterol control*			
LDL < 100 mg/dl†	2.45	63.7	
LDL ≥ 100 mg/dl†	1.39	36.3	
LDL < 70 mg/dl†	0.83	21.5	
LDL ≥ 70 mg/dl†	3.02	78.5	
*ACE inhibitor/ARB use*			
Yes	3.55	45.9	
No	4.19	54.1	
*Statin use*			
Yes	4.54	58.6	
No	3.21	41.4	
*Beta-blocker use*			
Yes	4.01	51.8	
No	3.73	48.2	
**Persons with Diabetes**	17.6		8.33 (0.39)
*Glycemic control*			
HbA1c <7	9.09	51.6	
HbA1c ≥7	7.69	43.6	
*Blood pressure control (JNC VII)*			
SBP <130 & DBP <80 mm Hg	8.95	50.8	
SBP ≥130 or DBP ≥80 mm Hg	8.68	49.2	
*Blood pressure control (2014 Hypertension guidelines)*			
SBP <140 & DBP <90 mm Hg	12.5	73.1	
SBP ≥140 or DBP ≥90 mm Hg	4.6	26.9	
*ACE inhibitor/ARB use*			
Yes	8.95	50.8	
No	8.68	49.2	
**Persons with Hypertension**	61.9		29.21 (0.77)
SBP <140 & DBP <90 mm Hg	41.1	69.2	
SBP ≥140 or DBP ≥90 mm Hg	18.3	30.8	
**Persons with Kidney Disease**	3.53		1.67 (0.13)
*Blood pressure control (JNC VII)*			
SBP <130 & DBP <80 mm Hg	2.1	60.0	
SBP ≥130 or DBP ≥80 mm Hg	1.4	40.0	
*Blood pressure control (2014 Hypertension guidelines)*			
SBP < 140 & DBP < 90mm Hg	2.7	81.8	
SBP ≥ 140 or DBP ≥ 90mm Hg	0.6	18.2	

*The US total population is limited to adults age 20 years and older with Stage A HF who do not have a clinical diagnosis of HF

^†^Note LDL only measured in subgroup of total population, proportions are based on the total of individuals with measured values

Abbreviations: HF, heart failure; CHD, coronary heart disease; MI, myocardial infarction; LDL, low density lipoprotein cholesterol; HbA1c, hemoglobin A1c; SBP, systolic blood pressure; DBP, diastolic blood pressure; ACE, angiotensin converting enzyme; ARB, angiotensin receptor antagonists; Statin, HMG-CoA reductase inhibitors; Beta-blocker, beta-adrenergic blocking agent

**Fig 1:**
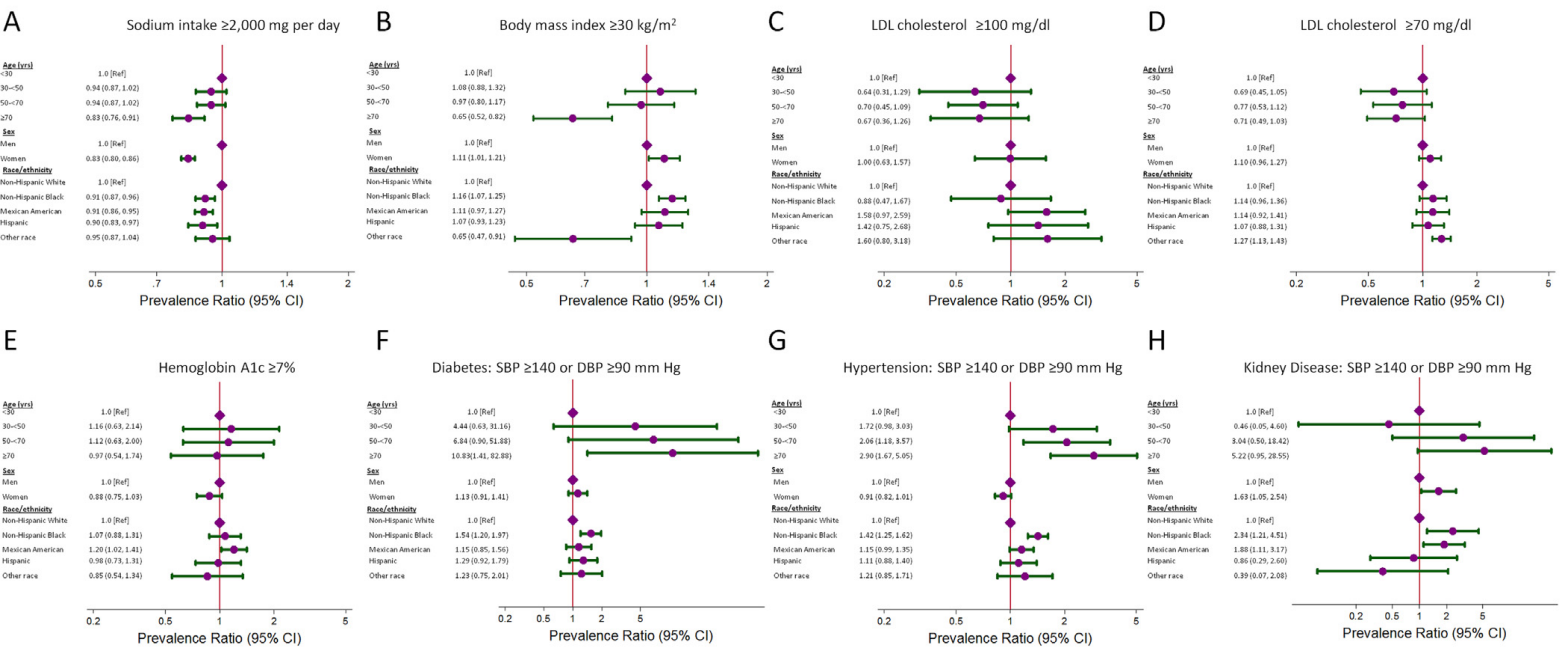
Demographic factors (age, sex, race/ethnicity) associated with poor risk factor control in US adults with stage A heart failure (HF). Factors examined were (A) sodium intake ≥2,000 mg/d in any adult with stage A HF, (B) body mass index ≥30 kg/m^2^ in any adult with stage A HF, (C) low density lipoprotein cholesterol (LDL) ≥100 mg/dL (≥2.59 mmol/L) in participants with a history of coronary heart disease or myocardial infarction, (D) LDL ≥70 mg/dL (≥1.81 mmol/L) in participants with a history of coronary heart disease or myocardial infarction, (E) hemoglobin A1c ≥7% in participants with a history of diabetes, (F) systolic blood pressure (SBP)≥140 mm Hg or diastolic blood pressure (DBP)≥90 mm Hg in participants with diabetes, (G) SBP≥140 mm Hg or DBP≥90 mm Hg in participants with hypertension, and (H) SBP≥140 mm Hg or DBP≥90 mm Hg in participants with kidney disease. Diamonds represent the reference groups. Circles represent the prevalence ratios. Horizontal capped lines represent the 95% confidence interval.

Fig 1 shows a stratified analysis of risk factors by age, sex, and race. Sodium intake was lower with increasing age. Women more commonly consumed <2,000 mg of sodium compared with men (PR: 0.83; 95% CI: 0.80, 0.86; *P* <0.001). With regard to race, non-Hispanic white participants were the most likely to consume ≥2,000 mg of sodium. BMI was also lower with age, while women and non-Hispanic blacks were more likely to have a BMI ≥30, with PR 1.11 (95% CI: 1.01, 1.21; *P* = 0.03) and 1.16 (CI 1.07, 1.25; *P* <0.001), respectively.

For LDL in those with CHD or a prior MI, there was a trend towards better control in participants >30 years (Fig 1). In US adults with a diagnosis of diabetes, Mexican-Americans were more likely to have HbA1c ≥7%. In the same group with diabetes, non-Hispanic black adults had a PR of 1.54 (95% CI 1.20, 1.97) for SBP ≥140 mm Hg or DBP ≥90 mm Hg and a PR of 1.30 (95% CI 1.10, 1.53) for SBP ≥130 mm Hg or DBP ≥80 mm Hg (S1 Fig). Uncontrolled hypertension, defined by SBP ≥140 mm Hg or DBP ≥90 mm Hg, was more common in participants aged 50-<70 years and ≥70 years. Non-Hispanic black participants also had a higher prevalence of uncontrolled BP (PR 1.42; 95% CI: 1.25, 1.62). Among participants with kidney disease, women (PR 1.63; 95% CI: 1.05, 2.54), non-Hispanic blacks (PR 2.34; 95% CI: 1.21, 4.51), and Mexican Americans (PR 1.88; 95% CI: 1.11, 3.17) were associated with SBP ≥140 mm Hg or DBP ≥90 mm Hg. When using the JNC VII blood pressure goals, age ≥ 70 years (PR 7.57; 95% CI: 1.14, 50.17) and non-Hispanic blacks (PR 1.70; 95% CI: 1.17, 2.48) were associated with having a SBP ≥130 mm Hg or DBP ≥80 mm Hg.

In US adults with Stage A HF, after adjustment for demographic and socioeconomic factors, income ≥$20,000 was associated with a sodium intake ≥2,000 mg while insurance status and education level was not associated with salt intake (Table 4). The prevalence of BMI ≥30 kg/m^2^ was higher in those with health insurance (PR 1.13, *P* = 0.05) and lower in those with some college education (PR 0.90; *P* = 0.04). In those with prior CHD/MI, income ≥$20,000/year and health insurance were inversely associated with LDL ≥100 mg/dL with PRs of 0.58 (*P* = 0.03) and 0.56 (*P* = 0.03), respectively. Having health insurance was also inversely correlated with uncontrolled blood pressure with PR 0.70 (*P* = 0.002). Table 5 shows socioeconomic factors that are associated with appropriate treatment for US adults with Stage A HF. Income ≥$20,000/year was positively associated with statin use among US adults with prior CHD/MI. Health insurance was associated with beta-blocker use in the same population. In contrast, education was not associated with medication use.

**Table 4:** Socioeconomic Factors Associated with Uncontrolled Risk Factors in US Adults with Stage A HF.

	Prevalence Ratio, 95% CI					
	Income ≥ $20,000		Possess Health Insurance		Education ≥ Some College	
	Model 1*	Model 2†	Model 1*	Model 2†	Model 1*	Model 2†
**All persons with Stage A HF**						
Sodium intake ≥ 2000mg/d	1.12 (1.07, 1.17)§	1.10 (1.05, 1.15)§	1.08 (0.97, 1.19)	1.04 (0.96, 1.14)	1.04 (1.00, 1.09)‡	1.03 (0.99, 1.07)
BMI ≥ 30 kg/m^2^	1.04 (0.93, 1.15)	1.04 (0.93, 1.17)	1.12 (1.00, 1.27)	1.13 (1.00, 1.27)†	0.91 (0.83, 1.01)	0.90 (0.82, 1.00)†
**Persons with CHD/MI**						
LDL ≥ 100 mg/dL||	0.54 (0.35, 0.84)§	0.58 (0.36, 0.94)‡	0.48 (0.32, 0.75)§	0.56 (0.34, 0.93)‡	0.94 (0.62, 1.43)	1.18 (0.79, 1.75)
LDL ≥ 70 mg/dL||	0.88 (0.75, 1.03)	0.86 (0.73, 1.02)	1.08 (0.81, 1.44)	1.15 (0.82, 1.62)	0.99 (0.88, 1.11)	0.99 (0.88, 1.11)
**Persons with Diabetes**						
HbA1c ≥ 7%	1.01 (0.87, 1.18)	1.03 (0.88, 1.20)	0.86 (0.73, 1.00)‡	0.89 (0.74, 1.06)	1.02 (0.87, 1.20)	1.01 (0.86, 1.19)
SBP ≥ 130 or DBP ≥ 80 mm Hg	1.10 (0.94, 1.29)	1.13 (0.96, 1.33)	0.92 (0.76, 1.12)	0.99 (0.80, 1.23)	0.97 (0.82, 1.15)	0.93 (0.79, 1.10)
SBP ≥ 140 or DBP ≥ 90 mm Hg	1.01 (0.75, 1.36)	1.05 (0.77, 1.42)	0.66 (0.47, 0.95)‡	0.66 (0.46, 0.97)‡	0.90 (0.70, 1.15)	0.91 (0.71, 1.18)
**Persons with Hypertension**						
SBP ≥ 140 or DBP ≥ 90 mm Hg	0.91 (0.78, 1.07)	0.95 (0.81, 1.10)	0.69 (0.56, 0.84)§	0.70 (0.56, 0.86)§	0.96 (0.82, 1.11)	1.00 (0.86, 1.18)
**Persons with Kidney Disease**						
SBP ≥ 130 or DBP ≥ 80 mm Hg	1.10 (0.75, 1.61)	1.05 (0.69, 1.60)	1.74 (0.85, 3.56)	2.01 (0.93, 4.37)	1.21 (0.84, 1.73)	1.19 (0.85, 1.68)
SBP ≥ 140 or DBP ≥ 90 mm Hg	0.94 (0.47, 1.87)	0.96 (0.42, 2.16)	2.38 (0.63, 8.97)	2.66 (0.59, 12.08)	1.23 (0.78, 1.94)	1.24 (0.71, 2.17)

*Model 1: Adjusted for age, sex, race

^†^Model 2: Model 1 plus adjustment for health insurance, education, income

^‡^ *P* ≤ 0.05

^§^ *P* ≤ 0.01

^||^Note LDL only measured in subgroup of total population, proportions are based on the total of individuals with measured values.

Abbreviations: HF, heart failure; BMI, body mass index; CHD, coronary heart disease; MI, myocardial infarction; LDL, low density lipoprotein cholesterol; HbA1c, hemoglobin A1c; SBP, systolic blood pressure; DBP, diastolic blood pressure

**Table 5:** Socioeconomic Factors Associated with Treatment among US Adults with Stage A HF.

	Prevalence Ratio, 95% CI					
	Income ≥ $20k		Possess Health Insurance		Education ≥ Some College	
	Model 1*	Model 2†	Model 1*	Model 2†	Model 1*	Model 2†
**Persons prior CHD/MI**						
ACE inhibitor/ARB use	0.99 (0.76, 1.27)	0.94 (0.72, 1.23)	1.64 (0.87, 3.09)	1.55 (0.79, 3.01)	0.93 (0.73, 1.19)	0.94 (0.73, 1.20)
Statin use	1.33 (1.13, 1.56) ‡	1.25 (1.07, 1.46)‡	1.61 (0.97, 2.66)	1.49 (0.89, 2.47)	1.05 (0.87, 1.26)	1.00 (0.82, 1.23)
Beta-blocker use	1.27 (1.00, 1.63) ‡	1.16 (0.88, 1.53)	2.32 (1.22, 4.43)‡	2.27 (1.09, 4.74)‡	0.99 (0.81, 1.21)	0.93 (0.77, 1.13)
**Persons with diabetes**						
ACE inhibitor/ARB use	0.99 (0.86, 1.14)	1.00 (0.87, 1.15)	1.07 (0.86, 1.33)	1.04 (0.83, 1.31)	0.90 (0.77, 1.05)	0.92 (0.77, 1.09)

*Model 1: adjusted for age, sex, race

^†^Model 2: Model 1 plus adjustment for health insurance, education, income

^‡^ *P* ≤ 0.05

Abbreviations: HF, heart failure; CHD, coronary heart disease; MI, myocardial infarction; ACE, angiotensin converting enzyme; ARB, angiotensin receptor antagonists; Statin, HMG-CoA reductase inhibitors; Beta-blocker, beta-adrenergic blocking agent

## Discussion

Based on a survey of US adults, one in three adults had been told by a health professional that they have a condition consistent with Stage A HF, representing approximately 75 million people. Similarly, in patients with known CHD or prior MI, only half are on medications shown to be beneficial for secondary prevention and to prevent progression to later stage HF. While having health insurance was inversely associated with uncontrolled LDL and BP, education level was not associated with improved control of any modifiable HF risk factors except BMI. Participants with lower income had higher cholesterol and were less likely to be on a statin, though reported lower sodium intake than higher income participants.

The ACC/AHA stages of HF characterize the progression of HF from pre-clinical to end-stage disease. For Stage A HF, the current guidelines recommend counseling, risk factor reduction, and control of concurrent diseases. Hypertension is the most common risk factor for HF, shown to have a population attributable risk of 39% in men and 59% in women.[34],[35] Men with hypertension have a higher lifetime risk of developing HF, compared to normotensive men.[36] Despite being diagnosed with hypertension, over 30% of Stage A HF patients during the surveyed period had blood pressure over the targets set by JNC VII and the 2014 Hypertension guidelines.[21,22] We found similar findings for patients with diabetes, another well-known risk factor for HF.[37],[38] Control of diabetes is an important predictor of new onset HF, with every 1% increase in HbA1c correlating to a 8–19% increase in HF incidence.[5–7] In addition to controlling hyperglycemia, tighter control of blood pressure is important to prevent HF and led to a 56% reduction in heart failure risk in those with diabetes.[8] Several studies have shown that blocking the renin-angiotensin system in persons with diabetes can decrease the incidence of HF, with losartan leading to 32–41% risk reduction.[9,10] In our population, only half of the participants with diabetes were prescribed an ACE inhibitor or ARB and only 51.6% had a HgbA1c <7.0.

Similarly, treating CHD and lowering cholesterol are important ways to prevent the progression from Stage A to clinical HF. In people with CHD without HF, simvastatin reduces the incidence of HF after 5 years of follow-up from 10.3% to 8.3%.[11] Reduction of LDL cholesterol in stable CHD with high doses of atorvastatin was also shown to reduce heart failure hospitalizations by 26%.[12] Unfortunately, only 21% of the study participants with CHD/MI achieved an LDL <70 mg/dL, likely in part due to only 58% being on a statin. The use of ACE inhibitors after an MI reduce the incidence of HF by 39–46% in those without HF.[13–15] In those intolerant to ACE inhibitors, ARBs are considered a reasonable substitute—but the evidence for ARBs preventing HF in patients with CHD/MI is less clear.[39],[40] While not tested in Stage A HF, metoprolol succinate helps to prevent remodeling in people with Stage B HF, or asymptomatic LV dysfunction.[16] Despite the evidence for preventing HF in those with CHD/MI, only about half are prescribed ACE inhibitors/ARBs or beta-blockers.

In addition to controlling comorbid disease, the ACC guidelines include encouraging healthy lifestyle for Stage A HF. In the Physicians' Health Study, healthy lifestyle habits including normal body weight (BMI < 25) were associated with lower lifetime risk of heart failure.[36] Compared to those with normal BMI, obese subjects had twice the risk of developing heart failure, with a graded relationship shown between BMI and HF incidence including those in the overweight category.[41] BMI also plays a role in the effect of dietary sodium on HF development, with sodium intake of >2,600 mg/day compared to <1,200 mg/day associated with a 1.43 relative risk for HF in overweight subjects.

HF is an expensive disease, and requires some degree of health literacy, self-care, and access to providers to prevent hospitalizations and poor health outcomes. Lower socioeconomic status (SES) is associated with a higher risk of incidence HF by 30–50%.[42],[43] Our results suggest that control of hypertension, diabetes, and BMI is not different in lower income subjects with Stage A HF. However, lower income was associated with not being appropriately treated for CHD/MI. This corresponds to previous studies of people with CHD showing lower rates of secondary prevention and revascularization in lower SES groups.[44],[45] While education level was not associated with control of comorbid diseases or medication use in Stage A HF, health insurance was associated with control of hypertension and cholesterol as well as appropriate medication use in CHD/MI.

This study has some important limitations that warrant discussion. First, our definition of stage A HF is limited. Due to the absence of echocardiogram data, we likely include a number of participants with stage B HF. Conversely, we were unable to include adults with prior exposure to cardiotoxic medications, another subset of Stage A HF. Second, we utilize self-reported physician or health professional diagnoses to identify stage A HF. While this ensured that at least at some point both the study participant and a health provider were aware of a HF-predisposing condition, we did not include subclinical cases. By not including subclinical cases, we likely underestimate the prevalence of Stage A HF in US adults. Finally, the NHANES are cross-sectional studies. As a result, we are unable to make causal inferences.

Despite these limitations, our study has several important strengths. The complex sampling design of the NHANES minimizes selection bias, representing arguably the best available mechanism for estimating disease prevalence in the general US population.[17] This is particularly important for studies such as this, in which the focus is on the prevention of more severe disease, such as late-stage HF. Furthermore, the comprehensive and high quality data assessments allows for a thorough examination of disease prevalence, risk factors, and socioeconomic characteristics of study participants.

In conclusion, there is a high prevalence of US adults with Stage A HF, many of whom are not being appropriately or adequately treated for their risk factors. With over 75 million people at risk for developing the lethal disease HF, primary care providers and patients need to become more aware of their risk factors and aggressively treat comorbid disease to prevent progression to symptomatic HF. Additionally, more research is needed to further understand the social and contextual factors associated with achievement of national guidelines for risk factor control and medication use. Finally, HF prevention efforts should focus on aggressive, evidence-based therapy of those with multiple uncontrolled risk factors.

## Supporting Information

S1 Fig Demographic factors (age, sex, race/ethnicity) associated with poor risk factor control in US adults with stage A heart failure (HF). Examined here are lower blood pressure goals for US adults with diabetes (A) or kidney disease (B), namely a systolic blood pressure (SBP)≥140 mm Hg or diastolic blood pressure (DBP)≥90 mm Hg. Diamonds represent the reference groups. Circles represent the prevalence ratios. Horizontal capped lines represent the 95% confidence interval.
(TIF)
