# Supplementary figures and images for: Stage A Heart Failure Is Not Adequately Recognized in US Adults: Analysis of the National Health and Nutrition Examination Surveys, 2007-2010
Source: PLoS One. 2015 Jul 14;10(7):e0132228. doi: 10.1371/journal.pone.0132228 (PMC4501734; doi:10.1371/journal.pone.0132228)

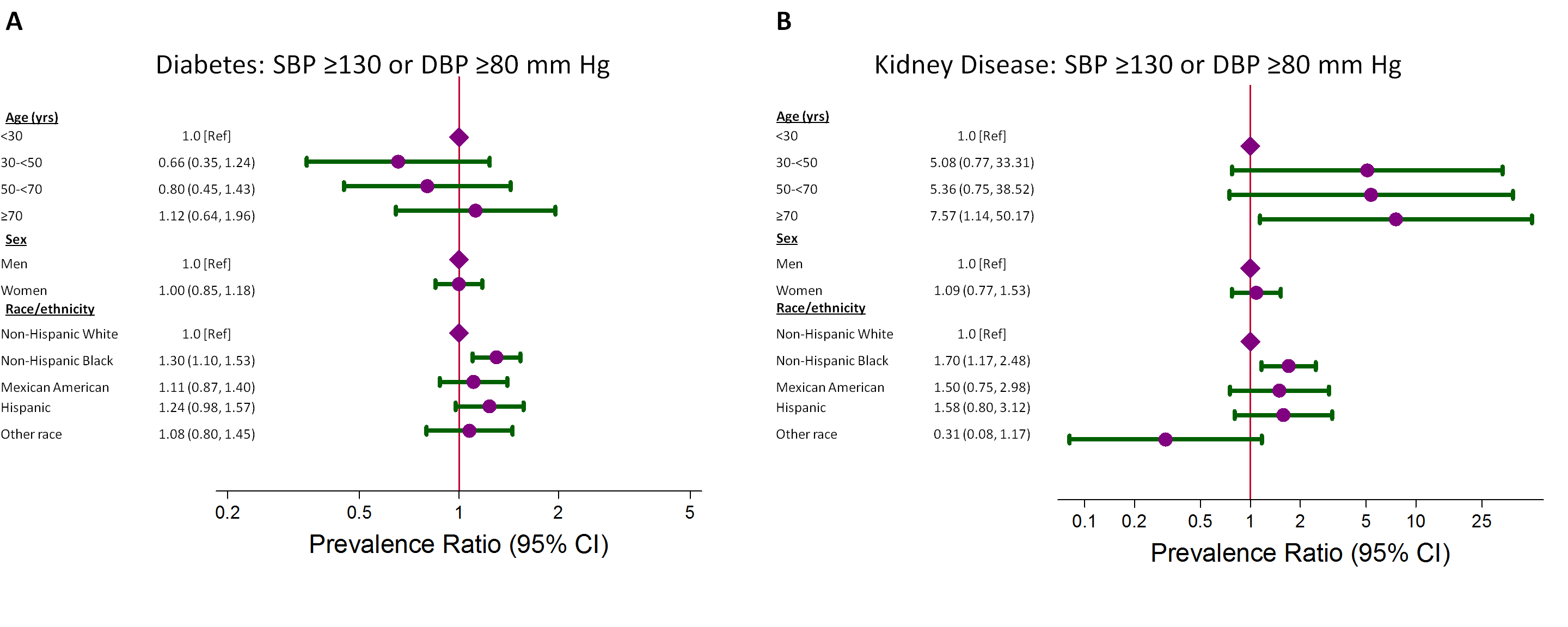

Supplement: S1 Fig — Examined here are lower blood pressure goals for US adults with diabetes (A) or kidney disease (B), namely a systolic blood pressure (SBP)≥140 mm Hg or diastolic blood pressure (DBP)≥90 mm Hg. Diamonds represent the reference groups. Circles represent the prevalence ratios. Horizontal capped lines represent the 95% confidence interval. (TIF) [file pone.0132228.s001.tif]
